# Commentary on: Are hepatitis B virus and celiac disease linked? HBV and Celiac Disease

**Published:** 2011-01-01

**Authors:** Kaushal Kishor Prasad, Arun K. Sharma, Chander K. Nain, Kartar Singh

**Affiliations:** 1 Department of Superspeciality of Gastroenterology, Post Graduate Institute of Medical Education and Research, Chandigarh, India

**Keywords:** Viral Hepatitis, Celiac Disease, Autoimmune Diseases, Hepatitis B Virus

Dear Editor,

Celiac disease (CD) is an autoimmune disorder characterized by small intestinal mucosal injury and nutrient malabsorption that affects patients with a specific genetic predisposition (HLA DR3-DQ2 and HLA DR4-DQ8) who are exposed to gluten, the major storage protein of wheat and similar grains [1][2]. Abnormal immune response to gliadin, genetic factors, and environmental factors play a role in the pathogenesis of CD. Infectious agents have been implicated in the pathogenesis of many autoimmune disorders. Transient infections or increased permeability of the mucosa may facilitate disease onset induced by the uptake of gluten peptides into a microenvironmental milieu in the small intestinal mucosa [3]. An environmental factor, such as an infectious agent, is thought to precipitate the disease via various pathogenic mechanisms, such as molecular mimicry, resulting in modulation of the host's immune tolerance [2]. Recently, the literature has reported on two patients who experienced an onset of CD after resolution of acute hepatitis B virus (HBV) infection [3]. CD has also been described in association with hepatitis C virus (HCV), rota virus, and adenovirus 12 as other immunologic manifestations of this infectious disease [4][5][6]. In the study by Leonardi and La Rosa (2010), which examined the possibility of HBV and its linkage with CD [7]. CD is more common in individuals with HLA DQ2 and HLA DQ8, and the literature has shown that these individuals have a lower response rate to HBV vaccination (50%-68%) than the general population (4%-10%; [8][9][10][11]). Recently, Ertem et al. (2010) examined the response to HBV vaccine prospectively in a group of CD patients and explored the potential link between CD and HBV-vaccine nonresponsiveness by studying shared HLA haplotypes. In their study they found that the response to HBV vaccine in celiac children who were compliant with a gluten-free diet (GFD) was not different from the response in a healthy population. CD may be one of the immune diseases associated with a high rate of HBV vaccine nonresponsiveness, but it might not be permanent, and treatment with GFD and compliance with the treatment may ameliorate the lack of responsiveness to the HBV vaccine in celiac children [12]. Therefore, nonresponsiveness to the HBV vaccine may be a sign of undiagnosed CD [9]. These findings provide useful information to reassess current vaccination strategies; in particular, revaccination is recommended during a controlled GFD [8]. Leonardi and La Rosa (2010)'s findings on the prevalence of CD in chronic HBV carrier patients is not conclusive given that only a few patients tested positive for IgG-AGA and IgA-AGA and none of the patients tested positive for IgA-EmA or IgA-tTGA. The prevalence of CD in the normal population of that area is not mentioned or compared in that study. Thus, the role of HBV as a triggering factor for CD seems to be a remote possibility. Activa tion of CD during interferon (IFN-α) therapy for hepatitis-virus infection is also a point of discussion. In the study by Leonardi and La Rosa (2010), none of the IFN-α-treated patients had any serological marker of CD [7]. CD has been epidemiologically associated with chronic hepatitis C and CD activation after the initiation of IFN-α in patients with HCV. However, a clear association of CD and HCV is lacking [13][14][15]. CD prevalence is not higher in patients with HCV. Routine screening of CD in HCV patients is not warranted; however, the presence of CD should be considered in the context of clinical deterioration during or after IFN- α therapy [15]. Plot et al. (2009) examined the association between serological evidence of past infection with Toxoplasma gondii, rubella virus, cytomegalovirus, Treponema pallidum, and Epstein-Barr virus and the coexistence of CD. The results implied that certain infections may generate an immunological environment that disfavors the future appearance of certain autoimmune conditions such as CD [16]. Therefore, understanding the relationship between infectious agents and autoimmune disorders is of utmost importance and may assist in the prediction, early diagnosis, and perhaps also the prevention of CD [2]. With the available evidence to date, it is difficult to establish a link between HBV, other viruses, and CD. Several isolated cases of infection with HBV and other viruses, which probably only reflect a fortuitous association with CD, have been cited in the literature. However, further studies are needed to explore whether a link between viral infection and CD really does exist.
